# Editorial: Molecular Dynamics at the Immunological Synapse

**DOI:** 10.3389/fimmu.2016.00632

**Published:** 2016-12-21

**Authors:** Andrés Alcover, Vincenzo Di Bartolo, Pedro Roda-Navarro

**Affiliations:** ^1^Lymphocyte Cell Biology Unit, Department of Immunology, Institut Pasteur, Paris, France; ^2^U1221, INSERM, Paris, France; ^3^Department of Microbiology I (Immunology), School of Medicine, ‘12 de Octubre’ Health Research Institute, Complutense University, Madrid, Spain

**Keywords:** immunological synapses, molecular dynamics, cytoskeleton, vesicular traffic, microscopy, fluorescente

**Editorial on the Research Topic**

**Molecular Dynamics at the Immunological Synapse**

The immunological synapse (IS) is a specialized cell–cell adhesion that mediates antigen acquisition, lymphocyte activation, and effector function. Seminal studies showed a structure composed of stable central and peripheral supramolecular activation clusters (cSMAC and pSMAC) organized at the interface of interacting helper T lymphocytes and B lymphocytes. The T cell receptor (TCR) and signaling molecules were found accumulated at the cSMAC, whereas the integrin LFA-1 and cytoskeleton components distributed at the pSMAC (1). The dynamics of these clusters at the IS was further tracked on the imaging plane by using antigen-presenting planar lipid bilayers (2). Currently, the IS is seen as a three-dimensional structure where signaling networks and components of the cellular machinery, including the endosomal compartment and the cytoskeleton, are polarized and reciprocally regulated to achieve proper T cell activation [Martin-Cofreces et al.; (3, 4)]. It has been also proved an important role of the IS in intercellular communication, being a local target for cytokine secretion and for the delivery of exosomes probably conveying important regulatory clues to antigen-presenting cells (APCs) (5, 6). The advent of new microscopy systems that improve the spatial and temporal resolution has shown the complex molecular dynamics at the IS. Early signaling is organized in dynamic microclusters at the periphery of the IS and, concomitantly, subsynaptic vesicles transport to these sites molecular components of signaling complexes (7–9). Much information is nonetheless missing about how this interplay between signaling and dynamic vesicular traffic and cytoskeleton is regulated.

This research topic (RT) contains 10 articles that cover different aspects of the molecular dynamics at the IS. Data are contributed on the spatial regulation of the signaling molecule Lck, an important molecular requirement for TCR triggering. With the assistance of super-resolution microscopy, Kapoor-Kaushik et al. provide a piece of original data to discuss how the spatial organization of Lck is regulated in activated T cells. Although the open conformation promotes clustering, signaling downstream the TCR further controls the spatial organization of Lck. Regarding the role of integrins in the triggering of the TCR and T cell activation, Hashimoto-Tane and Saito have recently demonstrated the existence at the IS of adhesion rings of integrins and focal adhesion molecules surrounding TCR-containing microclusters. This so-called microsynapse is proposed to support weak TCR activation *via* cell–cell local adhesion signals.

One novel and timely aspect discussed by Comrie and Burkhardt is how mechanotransduction, the transformation of mechanical forces into biochemical modifications, contributes to the TCR triggering and the intracellular signaling. The authors focus on the role of mechanical forces directed by filamentous actin (F-actin). The review contributed by Hivroz and Saitakis focuses on other mechanical clues that regulate T cell activation, including the effect of membrane protrusions and oscillations, cell mobility and spreading, the TCR engagement itself, or the engagement of LFA-1 during the IS formation.

Regarding the regulation of F-actin regulators, Ramirez-Munoz et al. propose that a local action of the cofilin activator Slingshot-1 at the IS might mediate an ultrasensitive/bistable response of the cofilin signaling module. This signaling module might then contribute to the specific and sensitive responses of naïve T cells and the more efficient and faster activation of antigen-experienced T cells.

The relevance of the cytoskeleton remodeling at the dendritic cell (DC) side is discussed by Benvenuti, who focuses her attention on the role of actin regulators, such as fascin and WASp, among others. The author also discusses about DC polarity and secretion induced by maturation stimuli. For example, the Cdc42-mediated polarization of the MTOC controls the delivery of IL-12 to the DC-T cell IS, a process mediated by VAMP7. Thus, it is envisaged that the activating signal three (inflammatory cytokines) is coupled at the IS to the activating signals one (TCR) and two (costimulation).

The dynamics of the endosomal compartment is discussed in the review by Onnis et al.. The authors revise the different Rab GTPases controlling the recycling routes targeting different receptors, such as the TCR and CXCR4, to the IS. They also highlight the recently noticed role of components of the intraflagellar transport system in controlling the traffic of the TCR to the IS downstream the centrosome polarization. This contribution poses the notion that IS and cilium constitute functional homologs. Important mechanisms of cell–cell communication are also described, including the trogocytosis and the local delivery of exoxomes and microvesicles.

Spatial organization of the IS also resembles the phagocytic cup, leading to the concept of the phagocytic synapse. Niedergang et al. remark this parallelism and discuss the organization, mechanism of assembly, and regulation of both structures. They pay attention to immune and phagocytic receptors, the interplay of the actin and tubulin cytoskeleton and the vesicular traffic. Discussion is provided about the role of soluble *N*-ethylmaleimide-sensitive factor attachment protein receptors and Rab GTPases in polarized vesicular traffic.

The structure and function of costimulatory and coinhibitory receptors upon the engagement of B7 molecules expressed on APCs are discussed by Brzostek et al.. They describe the function of CD28 and CTLA4 in the immune response, the regulatory role in the cytoskeleton dynamics and signaling and the distribution to the IS in effector and regulatory T cell.

Rocha-Perugini et al. discuss the role of tetraspanin-enriched microdomains in the local accumulation of receptors, adhesion molecules, and integrins at the IS. Associations are described between IS-located tetraspanins, several signaling molecules, and the actin cytoskeleton.

In summary, this RT highlights the fine-tuned molecular dynamics at the IS that allows proper T cell activation and effector functions. Methodological and technical advances in microscopy techniques improving spatial and temporal resolution are helping us to understand how the dynamics of the cytoskeleton and the endosomal compartment reorganizes micro and nanodomains of signaling complexes that, in turn, mediate lymphocyte immune responses. In addition, complementary biophysical approaches as well as the comparison with biological systems mentioned in this collection may provide useful hints to unravel the complexity of ISs.

## Author Contributions

PR-N wrote the first draft of the manuscript and updated the last version. AA and VB corrected and completed the initial draft.

## Conflict of Interest Statement

The authors declare that the research was conducted in the absence of any commercial or financial relationships that could be construed as a potential conflict of interest.
